# The Perceived Influence of AI on the Craftsmanship of Physicians: Qualitative Interview and Focus Group Study

**DOI:** 10.2196/93854

**Published:** 2026-09-23

**Authors:** Maran Lamberts, Wilco Brinkman, Harry van Goor, Michiel Tebbes, Merlijn Smits

**Affiliations:** 1 Research Group Industrial Design, Saxion University of Applied Sciences, Van Galenstraat 20, Enschede, Overijssel, 7511 JL, The Netherlands, 31 618210092; 2 CAOP Labour Research, Den Haag, Zuid-Holland, The Netherlands; 3 Department of Surgery, Radboud University Medical Center, Nijmegen, Gelderland, The Netherlands; 4 Department of Emergency Medicine, Slingeland Ziekenhuis, Doetinchem, Gelderland, The Netherlands

**Keywords:** artificial intelligence, craftsmanship, digital health, physicians, clinical competence, human-computer interaction, responsible design

## Abstract

**Background:**

Health care systems face rising demand and persistent staff shortages, intensifying pressure on the quality and sustainability of care. Artificial intelligence (AI) is increasingly being introduced to improve efficiency and decision support across clinical domains. While these tools promise operational gains, they can also reconfigure how physicians work, make judgments, and interact with patients, all elements of physicians’ craftsmanship. However, most research emphasizes technical performance rather than AI’s broader implications for physicians’ craftsmanship.

**Objective:**

This study aims to explore how physicians define craftsmanship in medicine and how they perceive AI to influence its professional and personal dimensions, with the goal of deriving practical principles for responsible AI design and implementation in hospital care.

**Methods:**

We conducted a qualitative, exploratory study in two phases (December 2024 to September 2025). Phase 1 involved semistructured interviews with 20 physicians from different hospital types and diverse specialties within the Netherlands. Phase 2 comprised two focus groups with physicians, physicians in training, hospital staff, policymakers, and AI developers during a national symposium, using an interactive, persona-based design to cocreate practical design principles.

**Results:**

Physicians described craftsmanship as their commitment to deliver the best possible care through human judgment, empathy, and contextual understanding. Perceived AI effects clustered in two areas: professional and personal dimensions. In professional dimensions, AI was seen to support workflow efficiency, documentation, data integration, and aspects of analytical reasoning, potentially freeing time for patient contact and reflection. Conditions for adoption included human-in-the-loop oversight, explainability, traceability, and AI literacy. In personal dimensions, empathy, contextual interpretation, and ethical judgment were viewed as inherently human and resistant to substitution. Concerns centered on de-skilling, less room for independent judgment, and threats to professional autonomy. Some variation was observed across specialties in how tasks and AI’s role were framed, reflecting their specific clinical contexts, but all shared the same core aim of delivering high-quality care. Based on the focus group discussions, the following design principles were identified that articulate how AI can be implemented in alignment with medical craftsmanship: consider a business case and strategic rationale; start from real clinical needs; let professional groups take the lead; design for contextual diversity; use user research for validation; design AI as supportive, not intrusive; safeguard autonomy and trust; cocreate with end users; learn across contexts; and use AI as a mirror for craftsmanship.

**Conclusions:**

AI seems to affect the conditions of professional craftsmanship and, thereby, indirectly the personal dimensions of it. This should be considered in design and implementation, while recognizing that continued interaction with AI may gradually reshape what craftsmanship itself comes to mean.

## Introduction

Health care systems globally are under increasing strain due to persistent staff shortages and a rising demand for care, threatening the sustainability and quality of medical services [1-4]. In response, artificial intelligence (AI) has emerged as a promising solution, offering the potential to enhance efficiency, support clinical decision-making, and improve patient outcomes across a range of medical specialties [2,5-7]. The integration of AI into clinical practice is accelerating, with applications ranging from diagnostics and treatment planning to patient engagement and workflow optimization [2,4,8-10]. At the same time, the rapid introduction of AI has raised widespread concerns among health care professionals about what this means for their role, with prominent scientific debates questioning whether AI might eventually replace parts of physicians’ work or primarily support it [11-13]. Despite growing interest in AI, research has largely focused on technical performance, with little attention to the actual impact on physicians’ professional identity or craftsmanship [1,2,7,14].

Historically, craftsmanship has been understood as the pursuit of optimal quality of work through the integration of expertise, ethical judgment, and dedication to service [15]. Sennett [16] captures this essence clearly, describing craftsmanship as “the desire to do a job well for its own sake.” Freidson [17] conceptualizes professionalism as an organizing logic that places quality of service and professional judgment at the center of work. Evetts [18] adds that professionalism is not only a set of values but also an institutional and organizational arrangement, through which professional authority, responsibility, and jurisdiction over work are structured. Building on these perspectives, Brinkman [19] synthesizes the work of Sennett, Freidson, and Evetts into 2 analytically distinct domains: professional craftsmanship, referring to the structural and organizational conditions for high-quality work, and personal craftsmanship, referring to the human qualities through which that work is realized. In this view, professional craftsmanship enables the enactment of personal craftsmanship.

**Figure 1.**
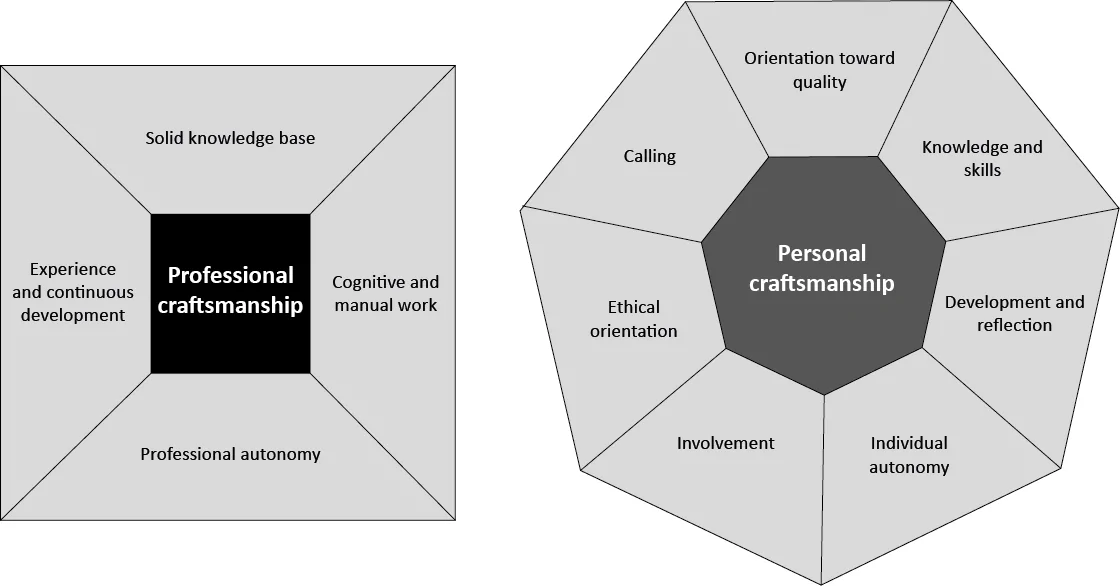
Conceptual framework of professional and personal craftsmanship, adapted from the work of Sennett [16], Freidson [17], and Evetts [18], as synthesized by Brinkman [19]. The framework distinguishes structural and organizational conditions of professional craftsmanship from individual human qualities that constitute personal craftsmanship and serves as the theoretical lens guiding this study.

As shown in Figure 1, craftsmanship is conceptualized as comprising distinct professional and personal dimensions. Professional craftsmanship is characterized by several structural features of a profession: the requirement of a (1) solid knowledge base, often built through specialized training and complex methodological reasoning; the integration of (2) cognitive and manual work, in which analytical judgment and practical action are inseparable; the presence of (3) professional autonomy, which provides room to make independent decisions in daily practice; and the reliance on (4) experience and continuous development as essential conditions for high-quality performance.

Personal craftsmanship, by contrast, refers to the qualities attributed to the individual professional. Key elements include (1) an orientation toward quality, the commitment to deliver the best possible work; (2) the accumulation of knowledge and skills through training, experience, and tacit understanding; (3) a continuous process of development and reflection; (4) individual autonomy in applying discretionary judgment; (5) a strong sense of involvement with one’s work, patients, and professional community; (6) an ethical orientation that prioritizes service over self-interest; and (7) a sense of calling, reflected in intrinsic motivation to remain dedicated to the profession even in the absence of external rewards.

In the medical profession, this distinction between professional and personal craftsmanship is particularly relevant. Physicians must meet formal requirements such as completing medical education, maintaining professional registration (in the Netherlands, the BIG register), and demonstrating continuous professional development, conditions of *professional craftsmanship*. Yet, their daily practice equally depends on *personal craftsmanship*: using experience and intuition, reflecting on one’s actions, and balancing ethical, relational, and evolving technical aspects of care.

In this study, we explore the perceived influence of AI on the craftsmanship of physicians working in hospitals through the lens of Brinkman’s craftsmanship framework. As the literature lacks a single, coherent conceptualization of craftsmanship [20], we build upon Brinkman’s synthesis [19] as an integrative framework. We focus on physicians working in hospitals in the Netherlands. Although AI is used across a range of health care professions and sectors, hospital physicians are uniquely positioned because they carry final responsibility for clinical decisions and exercise a high degree of professional autonomy. They are among the first to experience AI’s integration into diagnostic, procedural, and organizational contexts.

We start with studying how a diversity of physicians define their own craftsmanship and continue with understanding how AI may influence that understanding. By examining physicians’ perspectives, this study aims to contribute to a deeper understanding of how AI can be responsibly integrated into health care without compromising physicians’ craftsmanship. We define “responsible design and implementation” not as a technical or regulatory principle but as the development of AI that aligns with physicians’ own views on good quality of care and craftsmanship. This study is exploratory in nature and aims to identify how physicians perceive the influence of AI on different dimensions of craftsmanship. Rather than providing a comprehensive account of the mechanisms through which these changes occur, the study offers initial directions for understanding how AI may reshape craftsmanship and informs future research and design practices. In addition, this study includes a cocreative phase in which consortium members were involved as applied partners to translate findings into practical design principles. These stakeholders did not act as researchers in the analytical process but contributed domain-specific knowledge to ensure that the results are grounded in real-world practice and reflect multiple perspectives from the field.

## Methods

### Study Design

This study is part of the “AI als Arts” research (granted by Regieorgaan SIA, MV.KIEM.01.076) and took place between December 2024 and September 2025. Data collection took place between December 2024 and July 2025, followed by a period of data analysis and manuscript preparation from August to September 2025. It was led by the Saxion University of Applied Sciences research group Industrial Design and conducted together with Deventer Ziekenhuis (hospital), Medisch Spectrum Twente (hospital), Radboud University Medical Center (hospital), DearHealth (AI company), Health Valley (network organization), CAOP (Centrum voor Arbeidsverhoudingen Overheidspersoneel; consultancy & labor research), and Section Data & AI of the Netherlands Society of Emergency Physicians.

We used a qualitative, exploratory design consisting of 2 phases: semistructured interviews with physicians, followed by two focus groups. The interviews explored how hospital physicians understand craftsmanship and how they perceive AI’s influence on it, while the focus groups translated these insights into practical design principles. The focus groups were conducted to codevelop design principles for the responsible implementation of AI in hospital practice. The focus groups took place during a symposium at Deventer Hospital (the Netherlands) on June 13, 2025. This symposium was an initiative of the Dutch Knowledge Network for AI Implementation in Healthcare and was focused on the theme “Working smarter together: from university medical centres to small community hospitals.” The event brought together participants from across the Netherlands.

### Phase 1: Interviews

#### Participants

A total of 20 physicians were interviewed (see Table 1). Participants represented a diverse range of medical specialties and hospital types. To capture a broad range of perspectives, physicians from different specialties and hospital types were included (Table 1).

**Table 1.** Baseline characteristics of participating physicians (n=20) included in phase 1 of a qualitative interview study on the perceived influence of artificial intelligence on medical craftsmanship, conducted in hospital settings in the Netherlands between December 2024 and September 2025.

Characteristics	Values
Sex, n	
Male	15
Female	5
Age (in years), range	25‐65
Hospital type, n	
University medical center	4
Teaching hospital	11
Small community hospital	5
Specialism orientations, n	
Internal medicine	5
Surgical	9
Supportive	6

The participants’ contact information was provided through the research consortium, after which the researchers at Saxion contacted them through email. In-person interviews were recorded using a digital audio recorder. Online interviews conducted via Microsoft Teams were recorded verbatim using the platform’s built-in audio-recording functionality. The participants were interviewed from mid-December 2024 to late March 2025, with participation being fully voluntary with no additional incentive. The interviews lasted 45 to 60 minutes and were conducted either in person or virtually.

#### Materials and Instruments

The interview protocol was based on a pilot interview and consultations with domain experts in medical craftsmanship and the use of AI in hospital settings (see Multimedia Appendix 1 for the protocol). Because this study aimed to explore physicians’ own perceptions and lived experiences, no fixed definition of AI was provided during the interview. Instead, participants were invited to describe what they understood as “AI” in their clinical context and to refer to any technologies they associated with it. This approach ensured that all examples, whether technically accurate or not, reflected what physicians themselves perceive as AI, which is essential for understanding attitudes, expectations, and concerns.

The semistructured interviews were divided into 3 key parts. First, participants were asked about their background and what craftsmanship meant to them in their specific medical profession, including questions about core skills, values, and motivations in their daily work. The second part focused on their current experiences with AI in clinical practice, ranging from familiarity and observed use to perceived benefits and limitations based on their own definition of AI. The final part addressed expectations and concerns about AI’s future role, including its potential influence on craftsmanship.

#### Data Analysis

All interviews were transcribed using the transcription tool Amberscript (Amberscript BV). The transcripts were anonymized before analysis. Data were analyzed using Atlas.ti (ATLAS.ti Scientific Software Development GmbH) following a thematic analysis approach that combined inductive and deductive coding [21]. The exploratory nature of this study required an iterative and reflective process, led by two researchers who were both involved in the project. The researchers involved in the analysis brought different disciplinary perspectives to the study. The primary researcher has a background in human-centered design and qualitative research, while the second researcher has a background in psychology. These perspectives informed the coding process and interpretation of the data and were discussed during analysis to support reflexivity.

Initial coding followed an inductive and iterative process. Codes were developed from recurring patterns in the interview data and were informed by sensitizing concepts derived from earlier stages of the research. To establish analytic credibility and a shared understanding of the data, two researchers independently coded an initial subset of two interview transcripts. Coding decisions and interpretative differences were discussed in detail until consensus was reached, resulting in refinement and clarification of the coding framework.

The primary researcher subsequently applied the finalized coding scheme to the full interview dataset. Throughout this process, analytic decisions were documented and regularly discussed with the other researcher and, when needed, the full research team to support trustworthiness and analytic consistency. During this iterative process of coding and analysis, we continuously assessed whether new codes and themes emerged from the data. After 18 interviews, no new codes or conceptually relevant themes were identified. The two subsequent interviews did not yield additional insights but served to confirm and refine the existing codes and themes, indicating that thematic saturation had been reached.

To synthesize the findings, themes were developed by grouping related codes and identifying overarching patterns in physicians’ perceptions of craftsmanship and the impact of AI. The final themes were mapped onto the aspects of professional and personal craftsmanship.

### Phase 2: Focus Groups

#### Participants

Two focus groups were conducted, each with 15 participants, during a regional symposium on AI in hospital care. Participation was based on symposium attendance and self-selection into the workshop during the day. Each group included a heterogeneous mix of physicians, physicians in training, hospital staff with responsibilities for digital transformation, hospital policymakers, and AI developers. Because participants did not preregister individually for specific workshops, no additional individual demographic information was collected.

#### Materials and Instruments

The focus groups were designed as interactive sessions using structured exercises. In the first round, participants worked with 3 personas developed from the interview data (see Multimedia Appendix 2). These personas represented 3 nonclinical stakeholders involved in the design, policy, and implementation of AI in health care: an AI developer working at an external company, a policy advisor for digital health in a teaching hospital, and an implementation coordinator for digital innovations in a general hospital. Each persona faced distinct challenges in aligning AI with clinical workflows and professional values. The personas were constructed by synthesizing recurring frustrations, concerns, and collaboration challenges articulated in the interviews by physicians in relation to nonclinical stakeholders involved in AI development and implementation. To enhance credibility, the personas were discussed with experts (n=6) working in roles comparable to those represented, including AI developers and hospital-based AI implementation experts. These experts were recruited through the researchers’ professional networks and were consulted to better understand practical realities and to assess the recognizability and plausibility of the personas.

Participants were asked to empathize with the perspective of the physician and provide advice to these personas on how they could address their problems, needs, and dilemmas when collaborating with physicians in daily practice. Personas and related techniques such as role-playing are commonly used in focus group and participatory design research as effective instruments to enhance empathy and enable participants to take on perspectives other than their own [22].

For this interactive part, the Mentimeter software (Mentimeter AB) was used to allow each participant to provide input individually. Subsequently, participants worked in groups to reflect on the given advice (visible on a screen) and cocreate practical principles for how AI can best align with physicians’ craftsmanship, values, and daily clinical practice. For this, participants wrote down principles on a worksheet.

#### Data Analysis

All written materials and digital output of the focus groups were anonymized and imported into the collaborative digital workspace Miro (Miro, Inc) and treated as a single analytic dataset.

The primary researcher conducted an inductive thematic clustering of the data. Individual contributions were segmented into analytic units and iteratively grouped into thematic clusters, such as working from clinical practice and end-user needs, reflecting and continuous learning, integration with existing processes and collaborations, and business cases and strategic considerations.

Subsequently, thematic clusters were presented in a cocreation session with 7 members of the project consortium to translate them into concrete design principles for the responsible design and implementation of AI in hospital practice. The design principles were finally chronologically structured into an iterative step-by-step workflow.

### Ethical Considerations

This study does not fall under the Dutch Medical Research Involving Human Subjects Act and therefore did not require review or approval by a medical ethics review committee, in line with guidance from the Central Committee on Research Involving Human Subjects. The study was conducted in accordance with the ethical guidelines of Saxion University of Applied Sciences.

Participation was voluntary, and all participants provided written informed consent prior to participation. All data were collected and analyzed anonymously, with no directly identifiable personal information recorded. Data were stored securely and were accessible only to the research team. No compensation was provided for participation.

## Results

### Structure of the Results

In the first part of the results, we describe physicians’ definitions and current use of AI. We then structure interview results according to the dimensions of *professional* and *personal craftsmanship*. For each dimension, we first outline its definition based on Brinkman’s framework and then describe how physicians themselves articulated this aspect in their daily practice, including where their perspectives diverged from or extended the theoretical model. Each subsection then examines how physicians perceived AI to affect that specific dimension of craftsmanship. In the second part of the results, we describe focus group results: the design principles for the responsible design and implementation of AI in hospital care.

### Part 1: How Do Physicians Define Their Craftsmanship and How Do They Perceive the Influence of AI on Their Craftsmanship?

#### Physicians’ Definitions and Current Use of AI

When asked to define AI, physicians often described AI as a broad and ambiguous concept. Many participants indicated that they found it difficult to provide a clear definition, referring to AI as a “container concept” or “umbrella term” encompassing a wide range of technologies. Rather than offering formal definitions, physicians frequently described AI in functional terms, such as a system that supports or automates human tasks, or as a technology that performs certain activities faster or more efficiently than humans, as a physician said:

*There are so many different definitions of AI that it is hard to define it properly, but essentially it comes down to using computers to support or replace tasks such as decision making, texts, or images*.

[Radiologist]

When discussing current use, physicians referred to a wide range of AI-related applications. These included generative and supportive tools, such as large language models (eg, ChatGPT, Copilot, and Perplexity), which were primarily used for information retrieval, summarization, or writing support and were often applied informally or outside direct patient care.

Participants mentioned predictive and decision support applications, including *no-show* prediction models, discharge and readmission risk prediction tools, and triage support systems. Physicians had heard of these applications or were working with them as a part of pilot projects.

In addition, physicians referred to diagnostic AI applications, particularly in imaging-intensive domains such as radiology and pathology, including tools for image analysis, segmentation, and detection of abnormalities. Administrative and workflow-supporting technologies were also discussed, such as speech-to-text systems and process automation aimed at reducing documentation and registration burdens.

Overall, although awareness of AI applications was high, physicians reported limited active use of AI in routine clinical practice.

#### Physicians’ Definitions of Craftsmanship and Perceived Influence of AI

Across interviews, physicians depicted craftsmanship as a dynamic and fundamentally human practice in which knowledge, technical and social skills, reflection, and moral awareness come together. Central to this craftsmanship was a strong orientation toward quality: striving to deliver the best possible care within the realities of the health care system.

**Figure 2.**
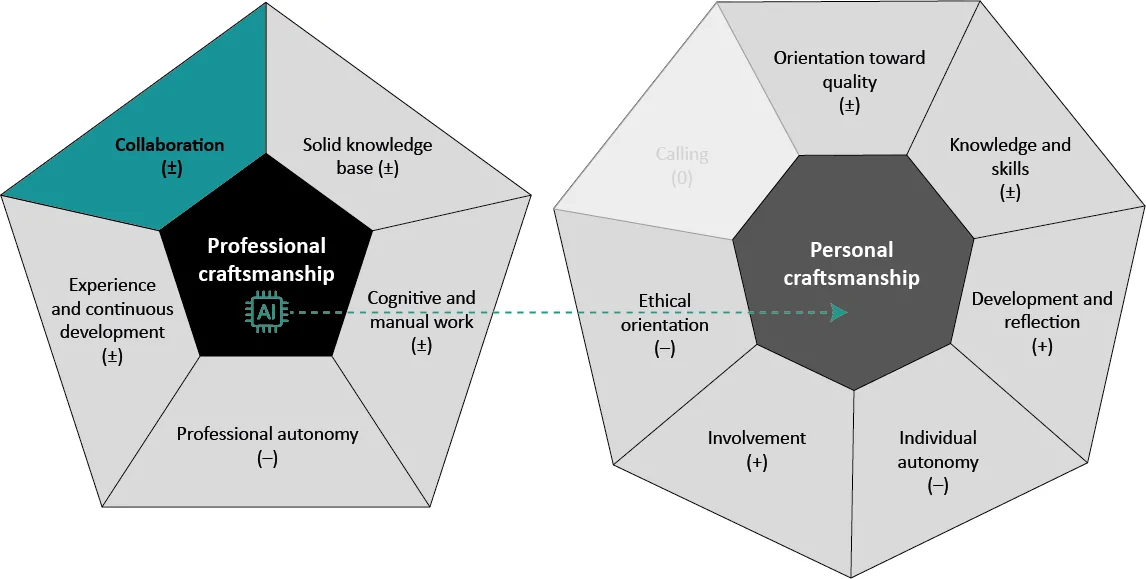
Physicians’ perceived direct and indirect influence of artificial intelligence on professional and personal dimensions of craftsmanship, based on 20 qualitative interviews conducted with physicians in hospital settings in the Netherlands (December 2024 to March 2025). Symbols indicate the predominant perceived influence: supportive (+), constraining (–), mixed (±), or absent (0).

Figure 2 visualizes how physicians currently perceived the influence of AI on different dimensions of craftsmanship. The perceived influence of AI on their craftsmanship was described as affecting the professional dimensions of craftsmanship (shown by the AI icon), while its perceived influence on personal dimensions was described as more indirect and mediated through changes in professional responsibilities, workflows, and decision-making processes (represented by the dashed arrow).

#### Professional Craftsmanship

Physicians unanimously acknowledged the core features of professional craftsmanship within the interviews.

##### Solid Knowledge Base (±)

In Brinkman’s framework, a solid knowledge base is a core element of professional craftsmanship. This is grounded in specialized education, formal training, and continual updating of expertise. Physicians echoed this view, describing their work as rooted in extensive theoretical knowledge complemented by domain-specific expertise that is built through training and accrues over time. In addition, they stressed that medical knowledge is inherently dynamic, making continuous learning a professional obligation rather than a personal choice. As one surgeon put it:

*For me, craftsmanship means being well informed about the latest developments and being able to explain clearly what the problem is and what the possible solutions are.*

[Surgeon]

Physicians described AI as reshaping the conditions under which a solid professional knowledge base is developed and maintained. AI was perceived to support access to and integration of medical information, while simultaneously introducing the need for new forms of knowledge, such as understanding the limitations, assumptions, and appropriate use of AI-generated outputs. At the same time, physicians expressed divergent views on whether all elements of the traditional medical knowledge base will remain equally necessary in the future. Some suggested that AI may reduce the need for certain forms of routine analytical or memorized knowledge, whereas others argued that a deep and comprehensive knowledge base remains essential to critically evaluate AI recommendations and to act safely when technology fails.

##### Cognitive and Manual Work (±)

Brinkman emphasizes that professional craftsmanship involves the inseparable integration of cognitive and manual work: analytical judgment and practical action are closely intertwined. In medicine, this means that thinking and doing cannot be meaningfully separated. Some participants highlighted the continuous interplay between decision-making and technical execution, as well as the translation of diagnostic reasoning into concrete treatment plans in daily practice. They also emphasized that interpretation, coordination, and technical action form a coherent whole. As one participant explained:

*Craftsmanship is often associated with manual skills. In rheumatology, we perform some procedures, such as ultrasound or joint injections, but far less than in surgical specialties. At the same time, our craftsmanship also lies in clinical reasoning and communication: examining patients, reaching a diagnosis, and discussing treatment options together in a safe and meaningful way.*

[Rheumatologist]

Physicians perceived AI as intervening directly in the organization of cognitive and manual aspects of medical work. AI was described as taking over or supporting specific cognitive tasks, such as data analysis, image interpretation, or protocol-based decision support, which are traditionally intertwined with practical action. In some contexts, this redistribution of tasks was welcomed as a way to reduce cognitive load and streamline workflows.

However, participants also noted that separating cognitive components from hands-on practice may alter the way these elements are coordinated in daily work. When AI systems prestructure decisions or recommendations, physicians may engage differently with subsequent actions, potentially changing how thinking and doing are coupled in practice.

##### Professional Autonomy (–)

Professional autonomy refers to the freedom that professionals are given to make independent decisions based on their expertise, especially in complex or uncertain situations. This autonomy is not absolute but embedded within professional standards, guidelines, and accountability structures. Physicians indicated that their profession offers space for professional autonomy, providing room to make independent decisions in complex care situations. At the same time, they emphasized that this autonomy only becomes meaningful when physicians are willing and able to exercise it, drawing on personal dimensions of craftsmanship. This was illustrated by one surgeon:

*Craftsmanship [...] is also about being able and willing to make choices. You can know a lot, but as a surgeon you must decide, take responsibility, and sometimes dare to choose.*

[Surgeon]

AI was most often perceived as constraining professional autonomy: algorithmic recommendations, embedded rules, and institutional expectations can narrow the discretionary space for clinical judgment. While physicians accepted AI for standardized or protocol-based decisions, they stressed that real-world cases often require contextual deviation. Several worried that treating AI outputs as normative may subtly shift decision-making authority and limit the room to justify, deviate from, or contest recommendations. Professional autonomy was therefore framed as a structural requirement for AI use.

##### Experience and Continuous Development (±)

Brinkman conceptualizes experience and continuous development as essential conditions for sustaining professional craftsmanship over time. Physicians strongly identified with this view. Experience was widely described as indispensable for becoming and remaining a competent physician:

*Craftsmanship is closely linked to experience. You complete your training and learn the basics, but real craftsmanship is ultimately developed in daily practice.*

[Urologist]

Physicians perceived AI as influencing the conditions under which professional experience is accumulated and maintained. On the one hand, AI was seen as offering new opportunities for learning, for example, by enabling feedback on decisions, supporting case review, or facilitating reflection through comparison between human and algorithmic outputs. In this way, AI could become part of the infrastructure supporting continuous professional development.

On the other hand, participants raised concerns that AI might reduce exposure to certain tasks or decision-making processes that traditionally contribute to experiential learning. If AI systems handle substantial parts of diagnostic reasoning or procedural planning, physicians may encounter fewer situations in which they actively build experience through practice.

##### Collaboration (±)

In addition to the elements of Brinkman’s framework, the participating physicians emphasized that collaboration should also be a central component of their craftsmanship. This was described as the ability and willingness to engage in effective multidisciplinary collaboration, coordinating and reflecting with other medical disciplines and teams:

*What I miss in the concept of craftsmanship is the multidisciplinary component. Every professional has a limited domain of knowledge and a specific set of skills, but true craftsmanship also lies in recognizing one’s own limitations and addressing them through collaboration within a multidisciplinary team.*

[Clinical technologist]

Respondents emphasized that AI will not reduce the need for collaboration. On the contrary, coordination, communication, and shared decision-making were seen as becoming more important, particularly in complex or time-critical situations. AI was described as potentially facilitating collaboration, for example, by supporting reflection or structuring information, but decision-making responsibility, mutual alignment, and decisiveness were framed as human tasks.

### Personal Craftsmanship

Physicians described personal craftsmanship as the human qualities that shape how professional work is carried out in practice. They described AI’s perceived influence on this personal dimension of craftsmanship as indirect, arising from changes in their professional context.

#### Orientation Toward Quality (±)

The essence of personal craftsmanship lies in the professional’s intrinsic drive to deliver the highest possible quality of work or service. Physicians echoed this notion, describing craftsmanship as the willingness for optimal quality of care. Quality was not limited to technical performance but also encompassed moral and relational dimensions: doing the right thing for the patient, the team or the system, and society. Three levels of quality orientation were identified: patient-level (individualized, value-based care), system-level (working within guidelines and resource limits), and societal-level (responsibility for equity and sustainability). As one surgeon explained:

*You just want to do it well, not only for the patient, but also for the team and for the profession itself*.

[Surgeon]

Overall, physicians anticipated that AI could indirectly support quality by reducing repetitive or administrative work, thereby creating more time for patient contact, reflection, and complex clinical reasoning. Some also noted that AI-supported data integration could contribute to more individualized decision-making at the patient level.

At the same time, physicians expressed concerns that excessive reliance on AI might undermine essential skills and, in turn, compromise care quality. Across interviews, participants emphasized that defining and safeguarding quality ultimately remains a human responsibility, requiring sensitivity to context and ongoing judgment. Maintaining this orientation toward quality was therefore closely linked to continuous education and AI literacy, ensuring that technological support does not replace, but informs, professional decision-making.

#### Knowledge and Skills (±)

Within personal craftsmanship, knowledge and skills refer to how individual physicians practically develop, embody, and apply expertise in situated clinical practice, rather than to the formal and collectively defined knowledge base that legitimizes the profession. Participants described these skills as experiential, tacit, and context-dependent, developed through repeated exposure, hands-on practice, and reflection rather than through formal instruction alone. As one surgeon remarked:

*You can’t learn this job from a book or video. You need flight hours.*

[Surgeon]

AI was perceived to influence this dimension indirectly. On the one hand, it introduces new personal competencies, such as maintaining critical oversight of algorithmic outputs and integrating AI-generated information into clinical reasoning. On the other hand, physicians cautioned that reduced engagement with certain tasks due to AI support may limit opportunities to exercise and refine experiential skills, potentially altering how personal expertise is developed and sustained over time.

#### Development and Reflection (+)

Within personal craftsmanship, development and reflection do not refer to the structured, collective arrangement of learning (experience and continuous development at the professional level), but to the individual’s reflective disposition: the willingness to critically examine one’s own actions, remain open to feedback, and adjust behavior accordingly. Participants framed this as a mindset that underpins integrity and everyday judgment, distinct from formal training structures. As one participant explained:

*If you’re not open to feedback or get angry about it, that’s where complaints come from, where patients become dissatisfied, and where things go wrong. Being trainable and accepting feedback, that’s just the basis of being a doctor*.

[Gynecologist]

When discussing AI, physicians generally viewed it as a potential aid to reflection. AI could support post hoc learning, for instance, by generating analyses or prompting comparison between clinical decisions and algorithmic outputs.

Overall, physicians considered reflection and self-development as enduring core elements of craftsmanship qualities that may be technologically supported but must remain firmly anchored in human responsibility and judgment.

#### Individual Autonomy (–)

Within personal craftsmanship, individual autonomy does not refer to the formal decision-making space granted to the profession but to the individual physician’s capacity to inhabit that space: exercising independent judgment, recognizing uncertainty, and taking responsibility for decisions in concrete situations. Physicians described individual autonomy as the ability to act at one’s own discretion based on training, knowledge, and experience. Autonomy was seen as knowing both the limits of one’s own expertise and when to seek support:

*Craftsmanship is knowing where your knowledge gaps are and where to find help. You don’t have to do it alone*.

[Clinical technologist]

Several participants stressed that context-sensitive judgment cannot be handed over to AI. While they acknowledged that AI might simulate aspects of communication or empathy, they were clear that tailoring care to the unique situation of an individual patient remains a human task.

Autonomy was therefore also used to draw a clear boundary for AI: algorithms may support protocol-based decisions but lack the capacity to judge when protocols no longer fit the situation.

#### Involvement (+)

Physicians described involvement as something that comes automatically with craftsmanship, an intrinsic sense of engagement with patients, colleagues, and the profession itself. This involvement was often framed in relation to taking or having time for being present, listening to patients, and staying up to date with knowledge to provide the best possible care. Several physicians linked this involvement to a sense of shared responsibility within their peer group.

However, participants also acknowledged that this deep level of engagement often comes at a personal price. The demanding nature of the profession was seen as both a source of pride and strain, as an emergency physician said:

*You end up rushing patients through the system, while you’d really like to give them more attention, precision, and care. But there’s simply no time, because you’re constantly checking boxes and logging things in the system.*

[Emergency physician]

Within this context, AI was perceived as a potential support for gaining more time for professional commitment during working hours. Physicians hoped that AI could reduce repetitive documentation tasks and administrative overload, allowing more space for genuine human interaction with patients. While many physicians viewed AI as a potential enabler of renewed patient engagement, some also warned that poorly designed systems could unintentionally distance them from patients, for instance, when screen interactions or automated reports take precedence over direct dialogue.

#### Ethical Orientation (–)

Physicians described ethical orientation as a fundamental and nonnegotiable component of craftsmanship, characterized by acting in the interest of patients, colleagues, and society rather than oneself. While Brinkman conceptualizes ethical orientation as an inner moral disposition, physicians in this study framed it more as ethically responsible action in practice, an expectation that applies universally within the profession. As one rheumatologist noted:

*When you look at ethically responsible behaviour, it’s really something overarching, something that applies to everyone. You can’t say that one doctor does it more than another*.

[Rheumatologist]

In the context of AI, physicians expected ethical judgment to become increasingly important as more cognitive and procedural tasks shift toward automated systems. Participants raised concerns about responsibility and accountability when decisions are influenced by AI, as well as uncertainty regarding the transparency and integrity of training data.

Several participants emphasized that ethical reflection should be explicitly incorporated into AI literacy and medical education.

#### Calling (0)

In Brinkman’s framework, calling refers to an inner drive or vocation that motivates professionals to commit themselves to doing good work. It concerns intrinsic motivation rather than formal requirements or organizational conditions. Across interviews, calling was rarely articulated as a distinct element of craftsmanship. Physicians typically described their motivation in pragmatic terms, interest in the field, educational and career opportunities, and treated it as a taken-for-granted part of being a doctor rather than something they reflect on explicitly.

*I never had a strong calling or a lifelong dream to become a doctor. I studied medicine because I found it interesting, and at some point you simply become a physician.*

[Dermatologist]

Unlike other dimensions, calling was not linked to AI by participants.

### General Insights

Physicians also articulated a crosscutting condition that shaped all discussions of AI: they consistently emphasized that AI should support, rather than replace, their clinical judgment. While they acknowledged that AI can inform decisions by processing information quickly or offering alternative perspectives, they stressed that final responsibility must remain with the physician. Maintaining a *human in the loop* was viewed as essential to safeguard professional autonomy and to ensure that contextual understanding, patient values, and individual circumstances continue to guide medical decisions. Physicians described this not as a preference but as a core condition for responsible AI use, grounded in their commitment to deliver high-quality care.

*When it comes to a protocol, a human can argue why they deviate from it. But an AI algorithm has no idea when it should or shouldn’t do that*.

[Radiologist]

### Part 2: What Does This Mean for Designing and Implementing AI Within Health Care?

#### Development of the Design Principles

**Figure 3.**
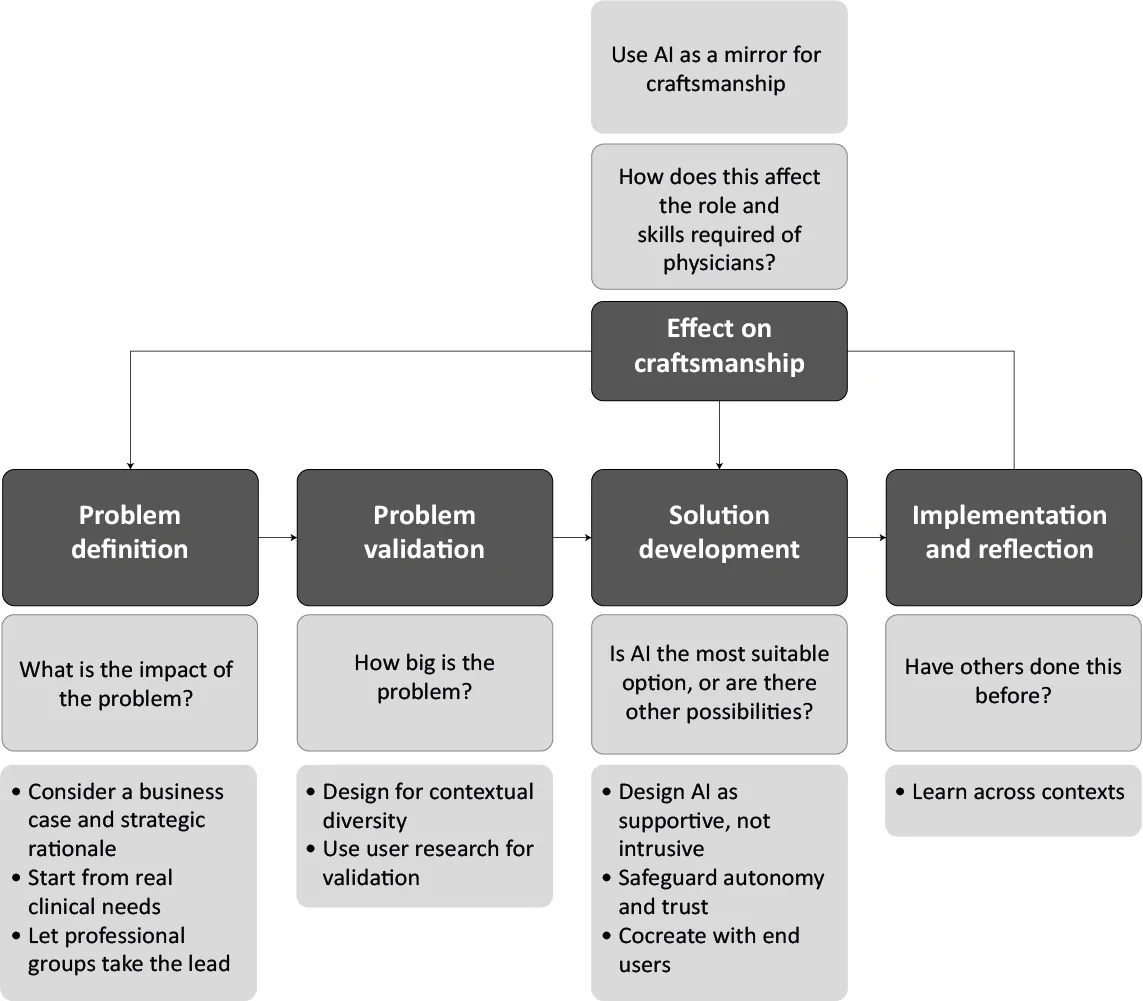
Iterative flowchart presenting design principles for the responsible design and implementation of artificial intelligence in hospital practice. The design principles were derived from two cocreative focus group sessions conducted during a national symposium in the Netherlands (June 2025) with physicians, physicians in training, hospital staff, policymakers, AI developers, and a cocreation session with members of the project consortium (July 2025).

The focus group discussions expanded on the interview findings by exploring how AI can be responsibly designed and embedded in medical practice to strengthen craftsmanship. The following design principles were derived: consider a business case and strategic rationale; start from real clinical needs; let professional groups take the lead; design for contextual diversity; use user research for validation; design AI as supportive, not intrusive; safeguard autonomy and trust; cocreate with end users; learn across contexts; and use AI as a mirror for craftsmanship. To support practical use, the design principles were synthesized into an iterative flowchart (Figure 3). The sections below describe the design principles and their position within the flowchart.

#### Problem Definition

The first step concerns identifying a real and clinically relevant problem that requires attention in daily work. Participants emphasized that AI design should always begin with a problem that physicians themselves recognize, rather than with a technology-driven initiative. Relevant design principles included:

- Consider a business case and strategic rationale: AI initiatives should be supported by a clear business case and aligned with broader organizational priorities to ensure feasibility and sustainability.
- Start from real clinical needs: AI should only be developed when there is a clear, shared problem that affects clinical workflow, patient care, or team functioning.
- Let professional groups take the lead: Physicians and their specialty groups should define which problems matter and what counts as quality in their domain, ensuring that the focus remains grounded in craftsmanship.

#### Problem Validation

This step involves examining whether the identified problem is genuine, widespread, and sufficiently significant to merit an AI solution. Participants stressed the importance of validating the problem across different roles, specialties, and hospital types. Relevant design principles included:

- Design for contextual diversity: Validation should consider the characteristics of different health care settings (academic, top-clinical, and peripheral) and specialties, as problems often manifest differently across domains.
- Use user research for validation: Interviews, observations, and focus groups were considered essential to understanding if the problem is shared, how it appears in practice, and whether clinicians agree on its urgency.

#### Solution Development

Once the problem is validated, stakeholders can explore whether AI is indeed the most suitable solution, or whether alternative approaches may be more appropriate. This stage requires a multidisciplinary workgroup to design and shape the solution. Relevant design principles included:

- Design AI as supportive, not intrusive: Participants preferred AI that complements craftsmanship by reducing administrative burden or offering analytical support, rather than replacing clinical reasoning or moral judgment.
- Safeguard autonomy and trust: Physicians should remain in control of decision-making. AI must be explainable, traceable, well-certified, and embedded in existing workflows to be trustworthy.
- Cocreate with end users: Developers, IT specialists, and clinicians should collaborate early, ensuring alignment with clinical processes, institutional infrastructure, and user needs.

#### Implementation and Reflection

This step involves introducing the solution into practice, evaluating its fit, and reflecting on its effect and workflow. This step is iterative, requiring pilots, adjustments, and continuous learning. The relevant design principle is as follows:

- Learn across contexts: Participants encouraged sharing success stories, failures, and lessons learned across hospitals to enable scaling without ignoring contextual differences.

#### Effect on Craftsmanship

This step concerns evaluating how the AI application influences the personal and professional dimensions of craftsmanship, both positive and negative. Participants emphasized that AI adoption should not only be assessed on performance or workflow efficiency, but also on how it shapes core clinical skills, judgment, and professional identity. The relevant design principle is as follows:

- Use AI as a mirror for craftsmanship: Physicians noted that AI can meaningfully support reflection by allowing clinicians to compare their own reasoning, diagnoses, or decisions with AI-generated suggestions. This “mirror function” helps identify which skills remain essential for safe, high-quality care and where new competencies must be developed. Participants viewed this reflective use of AI as an opportunity to strengthen craftsmanship rather than diminish it, if physicians remain in control of the final decision-making.

## Discussion

### Principal Results

#### Physicians’ Craftsmanship

Physicians defined craftsmanship as a dynamic practice. A practice that requires knowledge, technical and social skills, and (moral) reflection. The division between professional and personal craftsmanship, as defined by Brinkman [19], was recognized: our health system has established a professional context to work, to which the physician should relate.

All elements of professional craftsmanship were acknowledged as important by the physicians. Yet, they consistently wanted to add a component: collaboration, referring to the ability to engage in multidisciplinary collaborations with other medical professions. Collaboration remains underarticulated in classical craftsmanship accounts. Sennett [16], Freidson [17], and Evetts [18] primarily conceptualize craftsmanship as an individual pursuit, offering little elaboration on collaborative or team-based dimensions. Scholarship on medical professionalism, a term closely related to craftsmanship, however, does address collaboration. It states that good care arises from more than individual competence alone, but from the interplay between professional judgment, values, and collaborative practice [23-25].

For the components of personal craftsmanship, physicians recognized themselves well in all but one: calling. Calling, understood as an intrinsic or vocational drive, was not mentioned or recognized as belonging to craftsmanship. Although calling is often defined in the literature as an intrinsic motivation that provides meaning to work [26,27], physicians in this study appeared to distinguish it from craftsmanship, which they primarily associated with skills, knowledge, and ethical standards. Calling was instead framed as a personal motivation or a sense of vocation that may develop over time, rather than as an inherent feature of their craftsmanship. There is literature suggesting that physicians often initially experience medicine as a career path and only later, during or after training, come to experience their work as a calling [26]. In this sense, calling appears to function more as a background driver of professional engagement rather than as a constitutive element of craftsmanship itself.

Taken together, these findings suggest that while the framework offers a useful analytical lens, its dimensions do not fully align with how physicians conceptualize their craftsmanship, as collaboration emerges as a central component and “calling” appears to function more as a background motivation than as a defining element, pointing to the need for contextual reinterpretation.

#### Influence of AI on Craftsmanship

Overall, the craftsmanship framework served as a good reference point to discuss the influence of AI on medical practice. Most physicians referred to AI in broad, functional terms, frequently describing it as an “umbrella” or “container” concept encompassing a set of supportive and generative tools rather than a single, clearly defined technology. Given examples clustered into large language supportive tools, decision support applications, diagnostic applications, and administrative tools. Although no scientific literature is available on the current use of AI in Dutch hospitals, our results resemble a recent AI monitor, showcasing both generative AI and established clinical tools [28].

Where “dynamic” is already in physicians’ definitions of craftsmanship, AI shows the importance of the term. AI seems to directly influence the professional conditions under which physicians work: AI becomes a given in health care, requiring structural and organizational changes. Through its implementation, it might change the knowledge base needed to work and interact with AI, the ratio between cognitive and manual tasks, the autonomy that should be exercised, ways and needs for continuous development, and how professionals work together.

Through the reshaping of roles, physicians must relate to this new professional context, thereby indirectly influencing their personal craftsmanship. We encountered needs for redefining quality standards as AI becomes part of high-quality care delivery. Further, physicians should relate their knowledge and skills to AI: learning about AI might become part of the development of a professional, while learning tasks that AI has taken over might still be needed for when AI fails. The personal craftsmanship component of development and reflection stays in place, but again, AI might change how this is enacted as AI could be a mirror for action. Also, the need for autonomy seems to remain, but might become mediated by AI that creates a reliance of the physician on the model. Further, the involvement of a physician with their patients, colleagues, and profession seems to remain important, while being mediated in positive and negative ways. Finally, an ethical orientation seems to stay important and might even become more important since AI requires a new in-depth reflection itself.

So, our findings suggest that craftsmanship may evolve through the introduction of AI, requiring physicians to adapt to this changing context. Our results resemble recent literature, showing that changing working conditions require attention to ethical, organizational, and educational challenges [1,2,6,29,30]. By relating these insights to the concept of craftsmanship, an urgency to address them presents itself.

At the same time, the present study does not aim to fully explicate the mechanisms through which AI mediates the different dimensions of craftsmanship. Rather, the findings suggest that these influences are likely to be context-dependent, varying across medical specialties, organizational settings, and types of AI applications. For example, the way in which AI affects autonomy, knowledge development, or collaboration may differ substantially between clinical domains and technological use cases.

### Recommendations for Responsible AI

The flowchart starts with the first design requirement, establishing a business case and strategic rationale. The second design requirement then emphasizes starting from real clinical needs. Together, these requirements highlight that AI should only be considered when a clear clinical need is supported by a business case and strategic rationale, demonstrating its potential value for patients, professionals, or health care organizations. Although this is not AI-specific, technological innovations often fail as they are technology-driven, rather than needs-driven [31].

Identifying these needs cannot be done without physicians themselves. That is why the third design requirement referenced the need for professional groups taking the lead. Physicians emphasized the need for collective deliberation about where AI is acceptable, where it should be resisted, and how it can be responsibly embedded in practice to safeguard craftsmanship. Although literature emphasizes the importance of critically reflecting on the role of AI within each specialty [1,32,33], medical departments have so far rarely established systematic frameworks that define which aspects of their craftsmanship should be supported or delegated to AI.

The fourth design requirement addressed contextual diversity in design. Although our sample size is too small to establish differences in the perceived influence of AI on craftsmanship across specialties, gender, or hospital type, the results suggest potential variation that warrants further investigation. Within the literature, some of these differences have been studied. For example, adoption attitudes vary across specialties; surgical specialties have proven to be generally more inclined to use AI than nonprocedural fields [29,30]. Also, perceived usefulness, trust requirements, professional identity concerns, and readiness to adapt workflows differ per specialty [34-36]. Other differences have been described across demographic and organizational contexts. Previous studies suggest that perceptions of usefulness, trust requirements, and readiness to adapt workflows may vary by age, gender, and professional experience [37,38]. In addition, AI adoption and governance practices differ substantially across hospital types, with academic medical centers typically exhibiting higher levels of AI readiness and implementation capacity than smaller or nonacademic hospitals, reflecting differences in resources, infrastructure, and local context [39,40]. These different views on AI directly address the importance of the fifth design requirement to use user research to validate assumptions.

The sixth design requirement referenced the need to design AI as supportive rather than intrusive. In our study, we saw that physicians generally welcomed AI when it related to workflow optimization, documentation, and data integration. This resembles literature [2,41]. In contrast, physicians were cautious where AI touched personal domains such as empathy, ethical judgment, and patient communication, as they regarded these as inherently human.

The seventh design requirement emphasizes the need to safeguard autonomy and trust. Physicians in our study frequently addressed concerns on autonomy and potential skill erosion, echoing risks of overreliance and diminished clinical judgment. Such concerns are frequently reported in the literature [42-47]. Accordingly, we found that adoption was seen as conditional on clear guardrails, physician control, human-in-the-loop oversight, and transparency/explainability, also previously found by other scholars [48-50].

Design requirement eight is related to the need for cocreation. This is not only important at the start of a design process to identify needs, but also in later stages to ensure that the AI solution matches hospital-specific infrastructure and care pathways [51]. This iterative involvement of end users across the entire lifecycle of system development is consistent with principles of human-centered design, as formalized in ISO 9241-210:2019, which emphasizes continuous stakeholder engagement to ensure systems remain usable, relevant, and context-sensitive [52].

Within design requirement nine, the need for learning across contexts was stressed. This requirement aligns with evidence regarding the “pilotitis” syndrome, where a proliferation of isolated AI health intervention pilots rarely results in nationwide deployment or long-term institutional adoptions [53]. Health systems are increasingly encouraged to establish learning communities empowered to reflect on performance and explicitly discuss failure [54]. At the same time, as each health system operates within a unique context, successful scaling rarely follows a one-size-fits-all approach and instead requires innovations to be adapted locally to existing infrastructures and care pathways to become embedded in routine practice [31,54].

Finally, the tenth design requirement emphasizes the value of using AI as a mirror for craftsmanship following implementation. Physicians in the study noted that AI can augment their knowledge and support daily reflection. This finding aligns with literature indicating that AI-based decision support can function as a reflective tool, encouraging clinicians to critically evaluate their own reasoning and supporting reflective professional practice in everyday clinical work [55]. AI has been reported to support clinical efficiency and personalized care across various health care domains. However, trust among health care professionals remains a critical determinant for successful integration into clinical practice [56]. A systematic review indicates that such trust is primarily influenced by algorithmic transparency, adequate training and user familiarity, and alignment with existing clinical workflows [55]. In our study, physicians similarly emphasized that AI could only support reflection and augment craftsmanship when these conditions were met.

Where we saw in the study the dynamic nature of craftsmanship, mediated by AI in both positive and negative ways, AI might, over time, even reshape the definitions of craftsmanship. Thereby, it could alter what is understood as skilled work [57]. This implies that the evaluative frameworks currently used to assess the positive and negative effects of AI on craftsmanship may themselves be subject to change. In the future, it is necessary to keep track of how the framework of craftsmanship might evolve through AI and continue to use AI as a mirror for craftsmanship.

### Limitations and Future Work

This study was exploratory in nature, aiming to gain a first understanding of how physicians define craftsmanship and perceive the influence of AI on it. As such, several methodological choices were made to allow for openness and diversity in perspectives. Nevertheless, these choices also entail limitations. First, AI was intentionally discussed in broad terms, without specifying particular systems or applications. While this allowed for open exploration of physicians’ own interpretations, it also introduced variation and conceptual heterogeneity in how participants understood “AI.” As a result, fundamentally different technologies, such as generative language models, diagnostic systems, and predictive tools, may have been discussed interchangeably, potentially influencing the nature and specificity of the responses. This limits the ability to attribute findings to specific types of AI and should therefore be considered when interpreting the generalizability of the results.

Second, this study was conducted solely within the Dutch health care context, which represents a high-resource, digitally advanced health system with a specific professional culture and organizational structure. Although this setting allowed for in-depth exploration of physicians’ experiences with AI and craftsmanship, it may limit the transferability of our findings to other health care contexts. In particular, perceptions of AI, professional autonomy, and craftsmanship may differ in low-resource settings, health systems with different financing and governance structures, or contexts where digital infrastructure and regulatory frameworks are less developed. Consequently, our findings should be interpreted with caution when applied beyond similar high-income health care systems, and further research is needed to examine how the relationships between AI and craftsmanship manifest across diverse international and organizational contexts.

Third, we recruited participants to hear a variety of opinions from different specialties, types of hospital, and gender. There were, however, more men participating than women (15 male, 5 female) and also other characteristics (such as age or experience with AI) might affect opinions relating to craftsmanship and AI. Our conclusions are generalized. In future research, it might be interesting to investigate what characteristics affect opinions and how these vary between groups.

In addition, no detailed demographic data were collected for the phase 2 focus group participants, as these sessions were conducted within the context of an existing symposium. Although the focus groups included a broad range of stakeholders relevant to AI design and implementation in hospital care, the absence of individual demographic and professional characteristics limits the ability to assess group composition in more detail and to weigh the relative influence of specific perspectives on the resulting design principles. Furthermore, the translation of thematic findings into design principles involved a cocreation session with consortium members, which may have influenced the final formulation of these principles. Although these participants did not act as researchers but contributed practical expertise from different stakeholder perspectives, this step inherently involves interpretation and may reflect the perspectives and priorities present in the group. As a result, the findings from phase 2 should be interpreted as exploratory and indicative.

Lastly, we have not tested the design requirements in practice, nor do we have the exact knowledge of how to achieve them. For example, the requirement on the professional groups taking the lead is promising, yet challenging in practice. In future research, it is interesting to test the principles in a real design case and investigate how to create the right conditions for application.

Building on the exploratory nature of this study, future research is needed to further investigate how AI mediates the relationships between different dimensions of craftsmanship. In particular, more in-depth and context-specific studies are required to examine how these interactions unfold across medical specialties, organizational settings, and types of AI applications. By focusing on more clearly bounded use cases, future work can move beyond identifying affected dimensions toward a more detailed understanding of the mechanisms through which AI reshapes medical craftsmanship.

### Conclusions

In this paper, we have focused on the concept of craftsmanship to understand the impact of AI on the clinical practice of physicians in hospitals in the Netherlands.

Results show that AI changes working conditions (professional craftsmanship), to which physicians should relate (personal craftsmanship). Designers and health care professionals need to anticipate AI’s impact, because ignoring craftsmanship may make AI counterproductive to one of its core promises: reducing workload and relieving pressure on health care systems.

Achieving sustainable health care therefore requires looking beyond measurable outcomes, also considering the indirect effects of AI and placing craftsmanship central in the design of new AI technology.

## Supplementary material

10.2196/93854 Multimedia Appendix 1 Interview protocol.

10.2196/93854 Multimedia Appendix 2 Persona focus group.

## Abbreviations

AI: artificial intelligence
CAOP: Centrum voor Arbeidsverhoudingen Overheidspersoneel
