# Supplementary material for: The Perceived Influence of AI on the Craftsmanship of Physicians: Qualitative Interview and Focus Group Study
Source: JMIR AI. 2026 Sep 23;5:e93854. doi: 10.2196/93854 (PMC13600595; doi:10.2196/93854)
Supplement: Supplementary file 1 [file ai-v5-e93854-s001.pdf]

Multimedia Appendix 1 – Interview Protocol

# Interview protocol for exploring the impact of AI on physicians' craftsmanship

Preliminary remark: This interview protocol is the basis for the interviews to be conducted. The exploratory nature of the research means that the results of one or more interviews may lead to adjustments, deletions or new questions.

**Introduction to the Project (5 minutes)**

"*Thank you for your time and participation. This interview is part of a research project that focuses on understanding how artificial intelligence (AI) impacts the craftsmanship of doctors like yourself. With AI becoming more prevalent in healthcare, we want to explore what it means to practice craftsmanship in medicine and how new technologies like AI can impact this role. Your insights will help us to identify key factors for the responsible design and implementation of AI in healthcare, ensuring that it aligns with the needs and values of physicians.*"

[explain the structure of the interview, I will start with questions about your craftsmanship and what that means to you, and then about your experience and opinion about AI and what that means for you for your craftsmanship]

**[start recording]**

**Part 1: Understanding Craftsmanship for Doctors (15 minutes)**

1. **Introduction participant:**
   - Can you tell us something about yourself and your work? What specialism do you practice?
   - How much work experience do you have as a doctor?
   - What do you find most valuable or special about your profession?
   - What do you enjoy most about your profession?
   - What is the most important reason for you to do this work?
   - What motivates you in your work on a daily basis?
2. **Definition of Craftsmanship: Skills Arts**:
   - When you think of your own craftsmanship, what comes to mind?
     - *What do you mean by that, can you give an example? Why is [subject] a part of craftsmanship for you?*
   - What specific skills are important for your craftsmanship?
3. **Core competencies of the arts**:
   - What specific competencies do you think make the "craftsmanship" of the doctor?
     - *What do you mean by that, can you give an example? Why is [subject] a part of craftsmanship for you?*
   - When do you think you are doing your job well?

        - Asking questions; who determines whether the work is good
    - What makes you a good doctor?
        - Why? What does that show? Does that distinguish you from colleagues? Or not?

**Part 2: Current experiences with AI in healthcare (15 minutes)**

3. **Familiarity and Use**:
    - How would you describe artificial intelligence (AI)?
    - How familiar are you with AI technologies in your work?
    - How have you seen AI tools being applied in your work or in your department?
    - *Can you describe a recent example?*
4. **Perceived Value and Limitations**:
    - In what ways do you see AI contributing to your professional practice?
    - When does AI not help you in your work?

**Part 3: Expectations and Concerns about the Role of AI in Medicine (15 minutes)**

5. **Impact on Craftsmanship**:
    - What impact do you think AI will have on doctors' skills in the future?
    - Are there any specific skills or aspects of your job that you think AI can't or shouldn't replace?
    - What influence do you think AI will have on the competencies of the craftsmanship of doctors in the future?
6. **Professional autonomy and job satisfaction**:
    - When would AI reduce your job satisfaction as a doctor for you?
    - How do you feel about the possibility of AI taking over certain tasks that you currently enjoy?

      [name what participant has previously mentioned as pleasure in work]
    - When would AI increase your job satisfaction as a doctor for you?

**Closing and Closing Questions (5 minutes)**

- Is there anything else you'd like to add about AI and its potential role in healthcare?
- Do you have any questions about the research or the topics we discussed today?
