# Supplementary material for: The Perceived Influence of AI on the Craftsmanship of Physicians: Qualitative Interview and Focus Group Study
Source: JMIR AI. 2026 Sep 23;5:e93854. doi: 10.2196/93854 (PMC13600595; doi:10.2196/93854)

Tessa

AI developer at a startup for personalized care pathways

Position: AI developer at a health tech startup developing predictive models for personalized care pathways.

Focus: To design clinically relevant and safe AI that aligns with the EHR and supports physicians in their decision-making.

Challenges:

- Integration in hospital ICT is often a 'deal-breaker' → a lot of delays due to waiting times at ICT departments or lack of standardization.
- Gaining the trust of doctors requires clinical evidence, but that makes development processes slow and expensive (certification, studies, MDR).
- It is difficult to provide insight into how the model works (explainable AI), especially in generative applications.

"I want to make responsible AI that really works in practice, but you sometimes get completely stuck between data, legislation and hospital systems."

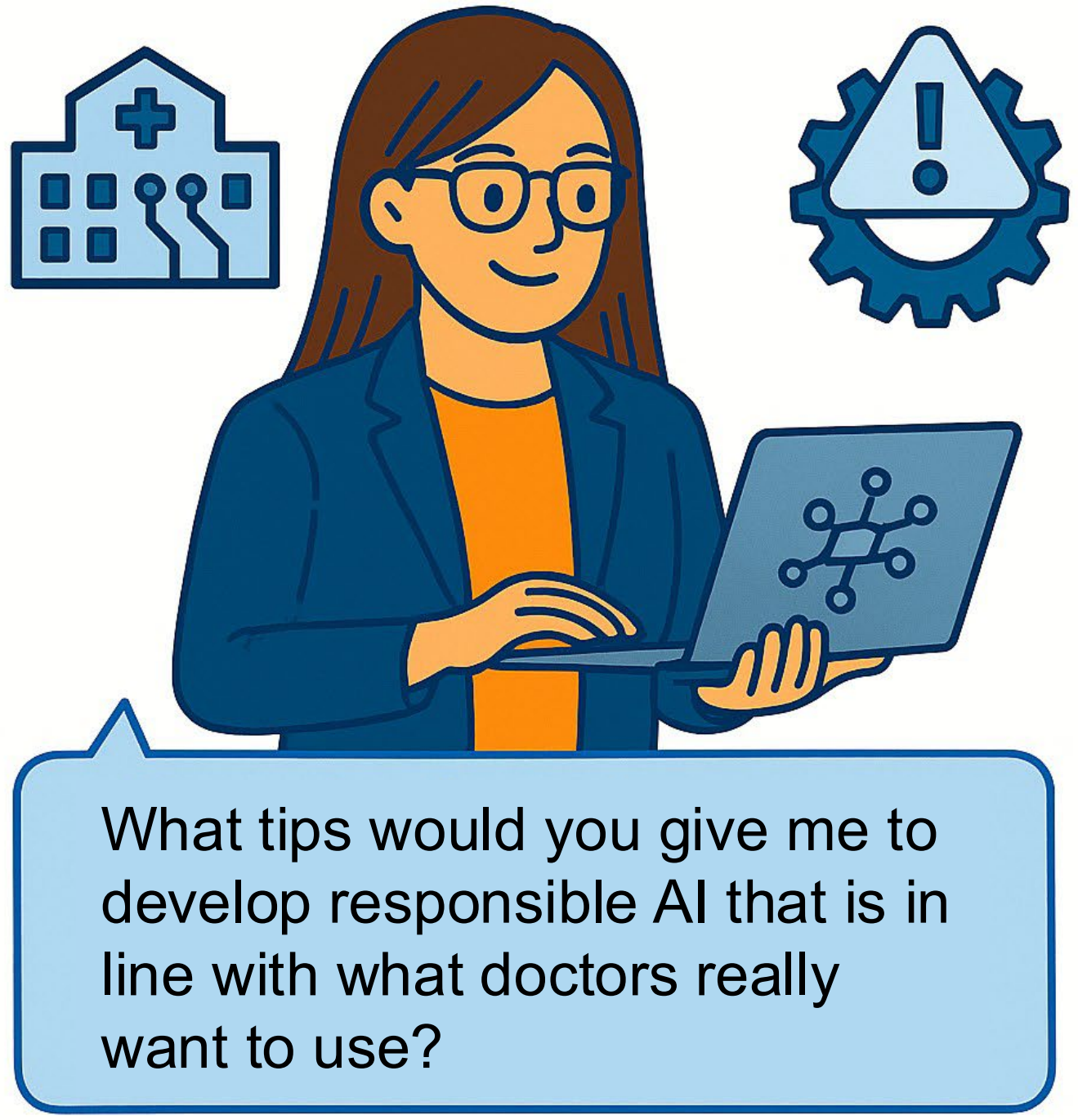

Martijn

Policy advisor digital care (Topklinisch)

Function: Policy advisor digital care, responsible for selection and procurement of technological innovations (incl. AI) within the hospital.

Focus: Safety, effectiveness and future-proofing of applications.

Challenges:

- Difficult to get a grip on the actual added value of AI; Evidence is often still in the research phase.
- The need for concrete returns (cost savings, time savings) delays adoption through lengthy validation processes with suppliers.
- Internal innovations are sometimes preferred because external development means: → certification obligation via MDR (expensive + long-term).
- Overloaded with proposals around AI that suggest employees to buy, high workload.
- Out of dissatisfaction, employees buy AI applications themselves, and therefore no longer have an overview of which applications are running in the hospital.

"Everyone is talking about AI, but I have to make choices based on evidence. If I can't prove that, I won't be able to buy it responsibly."

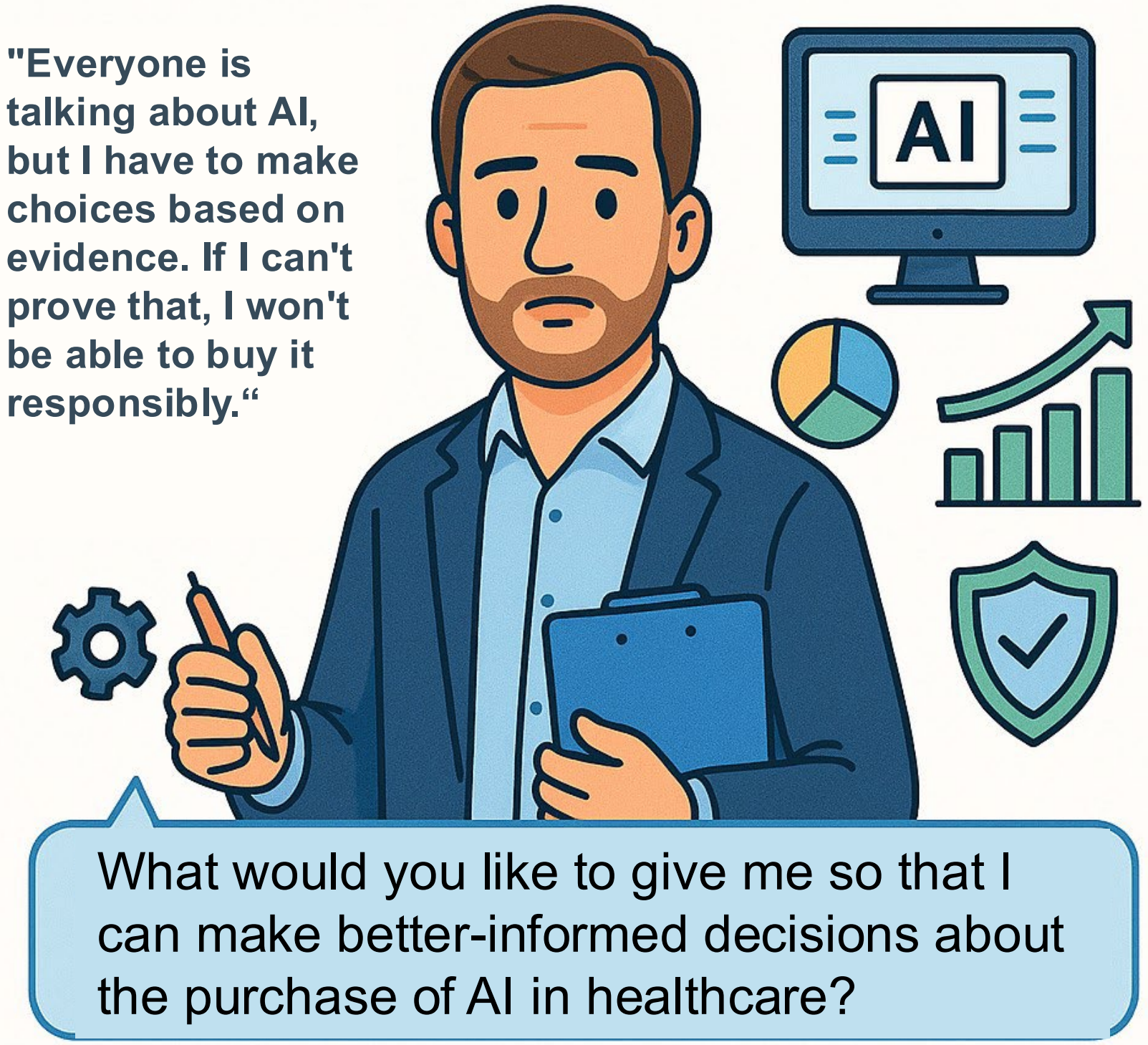

Fatima

Implementation coordinator digital innovations (Small general hospital)

Function: Responsible for adoption and supervision of AI applications within departments in the hospital.

Focus: Good connection to the workflow, user-friendliness, and support among healthcare staff.

Challenges:

- Implementation only succeeds if technology works intuitively and is easy to explain to users.
- Responsibility for training is often implicit in her position, without sufficient resources or mandate.
- No time or space for healthcare staff to practice or get used to new systems.

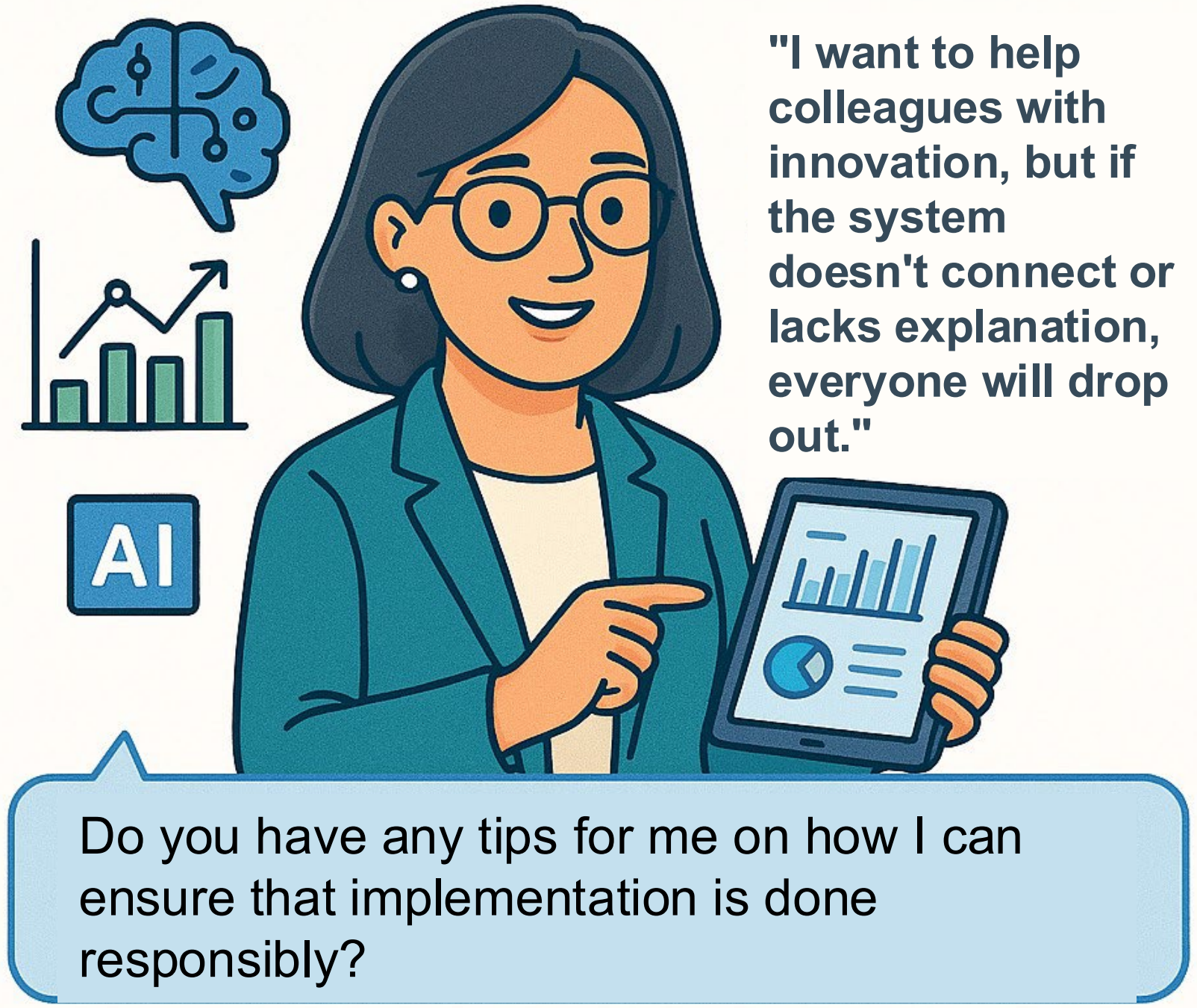

Supplement: Supplementary file 2 [file ai-v5-e93854-s002.pdf]
